# Distribution and Correlation of Ocular Surface Disease Index Scores in a Non-Clinical Population: The Karachi Ocular Surface Disease Study

**DOI:** 10.7759/cureus.9193

**Published:** 2020-07-15

**Authors:** Nauman Hashmani, Faizan Ghulam Mustafa, Muhammad Ali Tariq, Syed Farjad Ali, Fakiha Bukhari, Abdul Sami Memon, Sharif Hashmani

**Affiliations:** 1 Ophthalmology, Hashmanis Hospital, Karachi, PAK; 2 Ophthalmology, Shaheed Mohtarma Benazir Bhutto Medical College, Karachi, PAK; 3 Internal Medicine, Dow International Medical College, Karachi, PAK; 4 Surgery, Karachi Medical and Dental College, Karachi, PAK; 5 Ophthalmology, Karachi Medical and Dental College, Karachi, PAK; 6 Ophthalmology, Aga Khan University Hospital, Karachi, PAK; 7 Ophthalmology and Visual Sciences, Hashmanis Hospital, Karachi, PAK

**Keywords:** ocular surface, dry eye, dry eye disease, karachi, pakistan, prevalence

## Abstract

Introduction

There is increasing recognition of dry eye disease (DED) as a significant factor influencing quality of life in seemingly normal individuals. Our goal was to determine the distribution of Ocular Surface Disease Index (OSDI) scores in non-clinical individuals in Karachi, Pakistan.

Methods

We distributed OSDI questionnaires to subjects aged > 18 years with no active ocular complaint. Examiners were selected from various areas of the city to administer questionnaires to students and the general population. The OSDI score was grouped as per the following: normal (0-12 points), mild (13-22 points), moderate (23-32 points), and severe (33-100 points).

Results

We surveyed 2433 individuals with a mean age of 30.7±15.6 years. Additionally, the mean OSDI score was 22.4±18.7. To estimate prevalence, we used two OSDI score cutoffs: >13 (64.4%) and >22 points (43.6%). Statistical significance was found using multivariate regression in the following variables: age (p<0.001), contact lens wear (p<0.001), ocular allergies (p<0.001), hypertension (p<0.001), diabetes (p=0.003), and smoking (p=0.047). When graphing mean age against OSDI score, there was a large jump between the third and fourth decades; thereafter, there was a steady increase. Similarly, when plotting smoking, the score was steady until five years and then there was a sharp incline.

Conclusion

There was a high prevalence of DED in the studied population. Additionally, many systemic and ocular factors were associated with this disease.

## Introduction

The International Dry Eye Workshop (DEWS) II has defined dry eye disease (DED) as a multifactorial disease affecting both the ocular surface and the tear film in which increased tear film hyperosmolarity, ocular surface inflammation, and neurosensory problems can play a causative role [1]. The prevalence has varied throughout the world. It has ranged from 7% in the USA to 33% in Japan and Taiwan [2]. Additionally, a multitude of risk factors has been identified such as age, smoking, and contact lens wear.

Several tools have been created to screen for this disease effectively and efficiently. These include the Ocular Surface Disease Index (OSDI), Impact of Dry Eye on Everyday life (IDEEL) [3], and the Standardized Patient Evaluation of Eye Dryness (SPEED) [4] questionnaires. Of these, OSDI is a validated and quick method of evaluating DED, which makes it ideal for testing a large population [5].

To our knowledge, no study has observed the prevalence of DED and observed its trends in a large population in Pakistan. This is precisely the primary goal of this study. The secondary goals are to observe the association of the OSDI score with a variety of demographic factors.

## Materials and methods

This was a cross-sectional study conducted in the city of Karachi, Pakistan, to assess the symptoms of dry eyes using the OSDI (Allergen Inc, Irvine, Calif, USA). Additionally, we also assessed risk factors like contact lens use, smoking, surgery, and alcohol use. The Ethics Committee of Hashmanis Hospital approved this study according to the tenets of the Declaration of Helsinki. Informed consent was obtained from each participant.

Samples and questionnaire

Examiners were selected from various areas of the city to administer questionnaires to other students and the general population using convenience sampling. Subjects over the age of 18 years were included in the study. This study was conducted in residents with no active ocular problems. We excluded patients with evidence of ocular surface disease and known autoimmune conditions.

The questionnaire was divided into two parts. The first part asked about history including smoking history or contact lens use. Additionally, it inquired about their comorbidities like hypertension and diabetes. The second part was the OSDI questionnaire where subjects were asked to describe their symptoms over the previous week. The questionnaire was administered as a guided interview in two languages, English and Urdu.

The OSDI questionnaire assesses 12 items that evaluate both the symptoms of dry eye and its effect on the vision. There are three sections that evaluate visual complaints, symptoms, and any environmental triggers. The respondent is asked to list his complaints ranging from 0-4; 0 being “none of the time” and 4 equating to “all of the time.” The total score was calculated via the following formula: OSDI = ([Sum of all question scores*100]/[Number of questions answered*4)] [6].

The OSDI score was grouped as per the following: normal (0-12 points), mild (13-22 points), moderate (23-32 points), and severe (33-100 points) [7]. Grouping for the number of years smoking was done in the following way: < 1 year, 1-5 years, 6-10 years, 11-15 years, 16-20 years, and 20+ years.

Sample size

We used the openepi calculator (openepi.com) to determine the sample size. We used an estimated frequency of 33% [2], a confidence interval of 99%, and a design effect 1.0. The minimum sample size came out to be 587.

Statistical analysis

All statistical analysis was performed using the Statistical Package for the Social Sciences (SPSS v23; IBM Corp., Armonk, NY). Descriptive statistics were used for calculating mean and standard deviations. Prevalence was determined with four different methods: an OSDI of >13 and >22 and those with and without ocular factors. A Pearson correlation test, independent t-test, and multivariate regression were used for the various variables. Graphs were created using Microsoft Excel (Microsoft Corp., Redmond, WA). A p-value of < 0.05 was considered significant.

## Results

General characteristics

We surveyed 2433 individuals with a mean age of 30.7±15.6 years. Additionally, the mean OSDI score was 22.4±18.7. The rest of the data can be seen in Table 1. The prevalence of dry eye with this data set came out to be 64.4% (cut-off 13), and 43.6% (cut-off 22).

**Table 1:** General characteristics *Excluded patients with a history of ocular surgery, contact lens use, and ocular allergies, **OSDI=Ocular Surface Disease Index

	All data	After exclusion*
Total	2433	1385
Male/Female	813/1620	556/829
Age	30.7±15.6	27.1±9.3
OSDI score**	22.4±18.7	17.9±16.1

**Figure 1:**
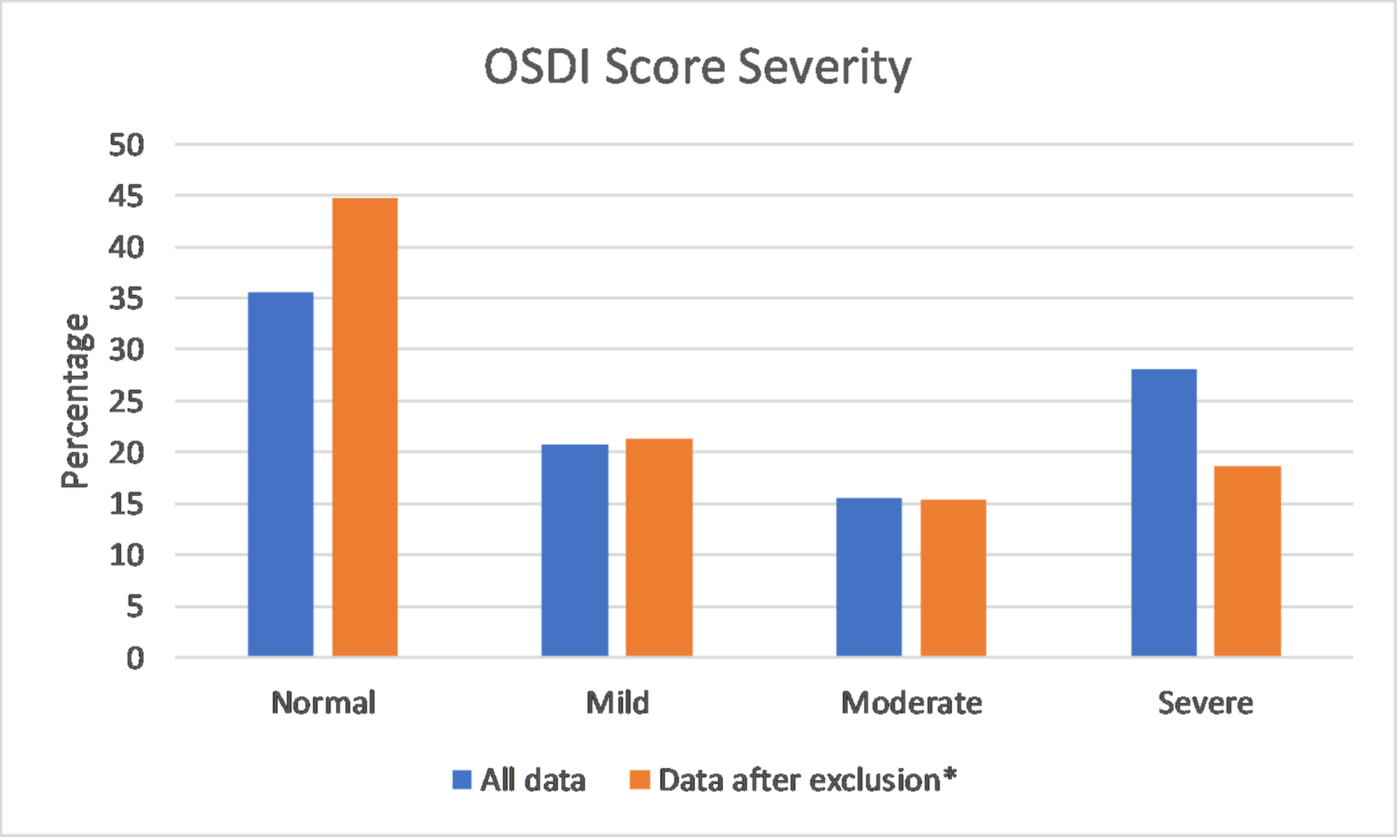
Distribution of OSDI scores *Excluded patients with history of ocular surgery, contact lens use, and ocular allergies OSDI=Ocular Surface Disease Index

We then repeated this analysis with the exclusion of patients with the following: a history of ocular surgery (n=139), contact lens use (n=601), and ocular allergies (n=391). The prevalence using this data came out to be 55.3% (> 13), and 33.9% (> 22). The distribution of the OSDI score can be seen in Figure 1.

Variables

Statistical significance was found in the following variables: age (p<0.001), contact lens wear (p<0.001), ocular allergies (p<0.001), hypertension (p<0.001), diabetes (p=0.003), and smoking (p=0.047). The rest of the data can be seen in Table 2.

**Table 2:** Effect of various variables on the OSDI score *SD=standard deviation, OSDI=Ocular Surface Disease Index

Variable	Number	Mean	SD	Monovariate P-Value	Multivariate P-Value
Age	2433	30.7	15.6	<0.001	<0.001
Gender				0.079	0.528
Male	813	23.4	18.9		
Female	1620	22.0	18.6		
Male > 45 years	134	34.5	17.3		0.321
Female > 45 years	246	37.8	17.4		
Contact lens	587			<0.001	<0.001
Yes		25.5	19.8		
No		21.5	18.3		
Smoking	329			<0.001	0.047
Yes		28.1	20.0		
No		21.6	18.4		
Steroid use	123			<0.001	0.304
Yes		35.3	20.2		
No		21.8	18.4		
Alcohol	76			0.021	0.633
Yes		28.8	22.7		
No		22.2	18.6		
Systemic allergies	446			<0.001	0.255
Yes		28.3	20.6		
No		21.1	18.0		
Ocular allergies	390			<0.001	<0.001
Yes		36.3	20.0		
No		19.8	17.3		
Ocular surgery	120			<0.001	0.189
Yes		32.4	19.9		
No		22.0	18.5		
Hypertension	383			<0.001	<0.001
Yes		35.7	18.7		
No		20.0	17.6		
Diabetes	139			<0.001	0.003
Yes		36.6	16.7		
No		21.6	18.4		

**Figure 2:**
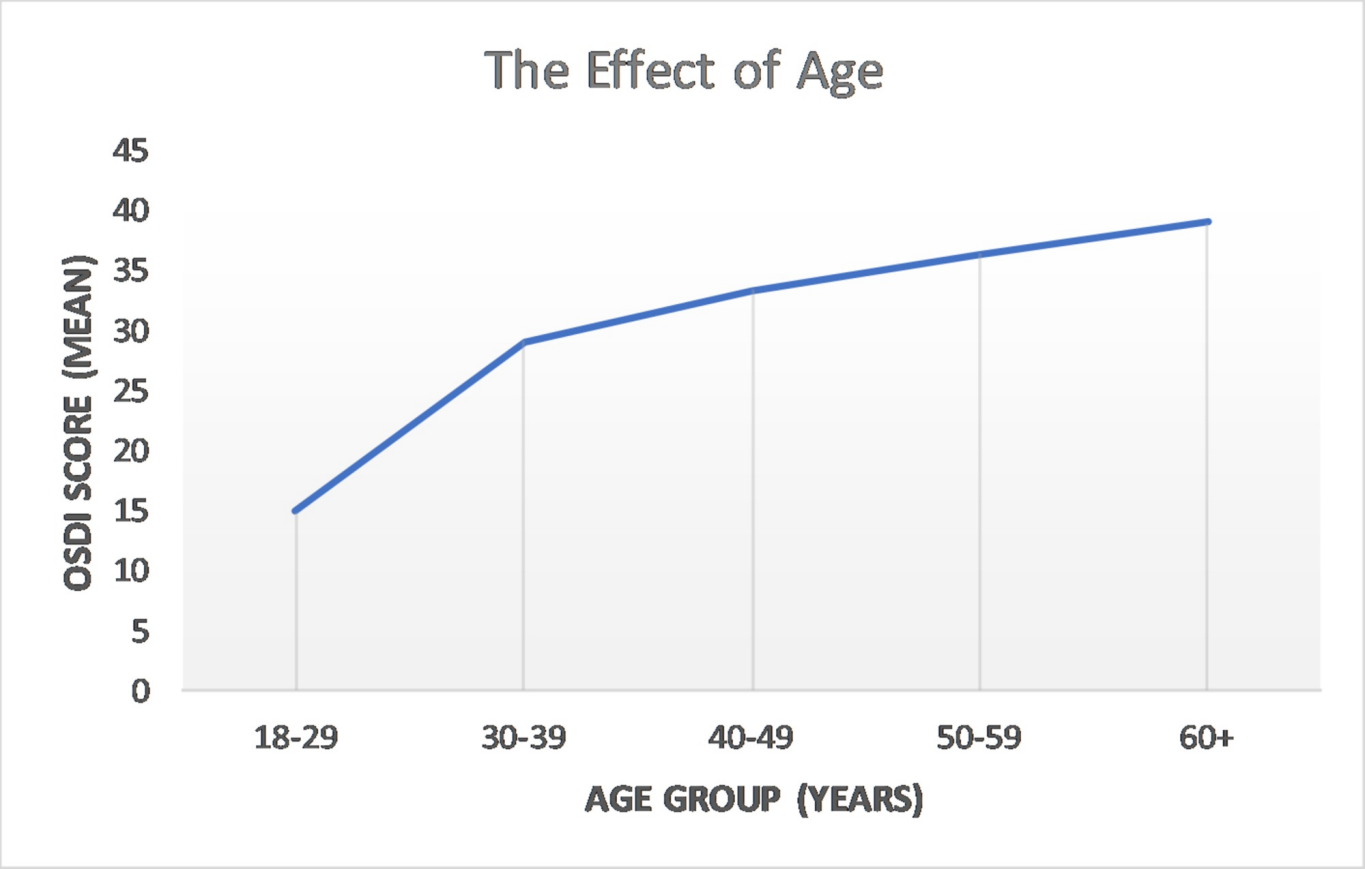
The effect of age on the OSDI score OSDI=Ocular Surface Disease Index

**Figure 3:**
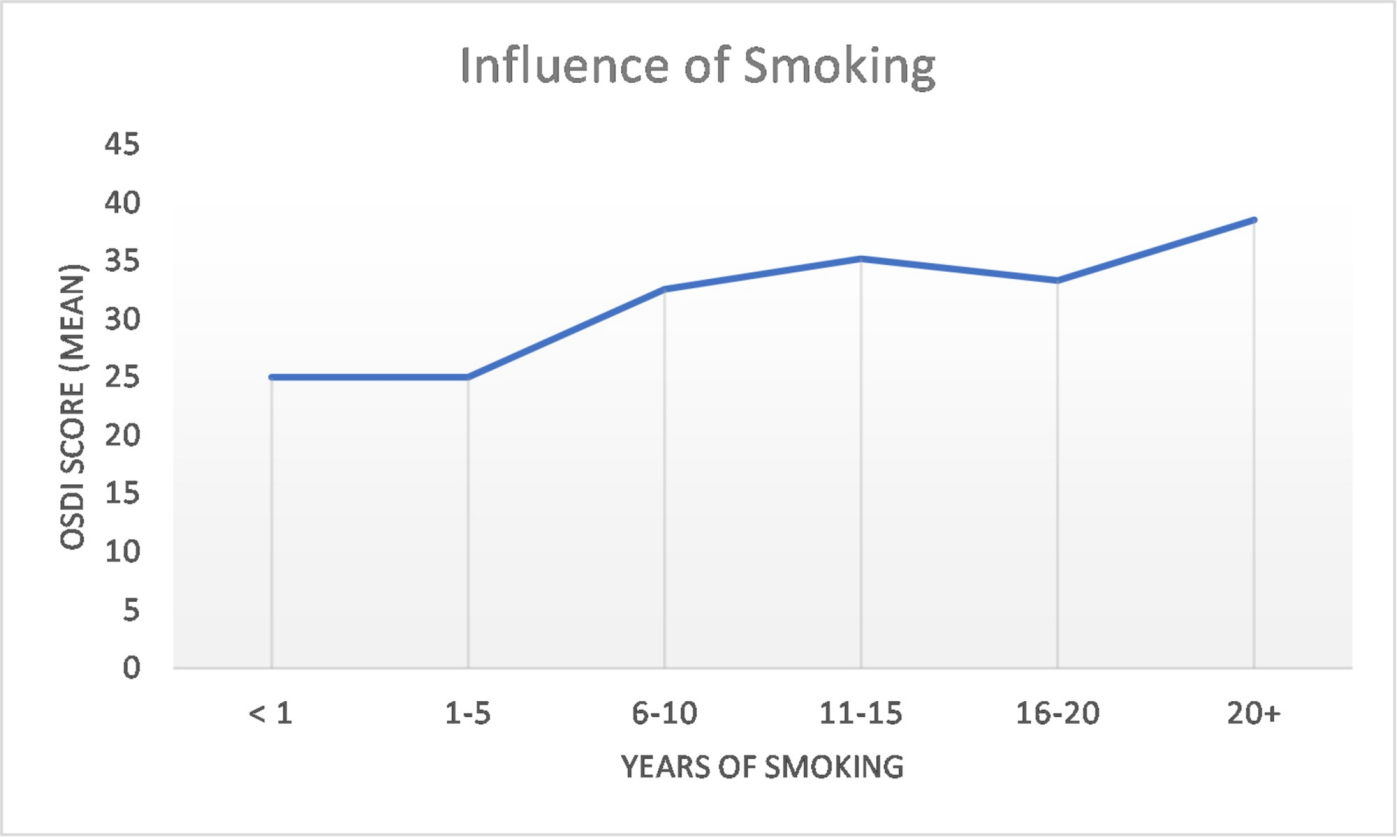
Influence of length of smoking on the OSDI score OSDI=Ocular Surface Disease Index

The effect of age on the mean OSDI score has been plotted in Figure 2. The greatest jump comes between the third and fourth decades and then there was a steady increase. Figure 3 shows the mean OSDI score plotted against the number of smoking years (p<0.001). The score remains steady until up to five years of smoking and then sees a sharp increase.

## Discussion

We performed a cross-sectional analysis using the OSDI questionnaire to understand the distribution of dry eye symptoms across a non-clinical population. Additionally, we observed the associations and trends of several factors that could possibly influence this disease.

There is a large variation in the prevalence of DED across the world. Studies in India [8], Jordan [9], France [10], and Iran [11] utilized the OSDI with a cut-off score of 20-22 points to calculate prevalence. They reported 32%, 59%, 39.2%, and 18.3%, respectively. Singapore used the McMonnies questionnaire to report a prevalence of 12.3% [12]. Numerous other studies have reported their own data with custom questionnaires and clinical signs; we are not discussing their prevalence as these are not directly comparable. Additionally, it is important to note that the first three studies used a high cut-off of 22 points to maximize sensitivity [13].

Therefore, we have reported values with a minimum value of 13 (mild) and 22 (moderate), as the true prevalence likely lies between these estimates. Furthermore, we subdivided our population further into those without ophthalmic factors affecting the ocular surface and those with. Therefore, we have reported a total of four prevalence values. The true prevalence lies between 33.9%-64.4%, depending on the criteria employed.

Many studies have linked the female gender as a risk factor for DED [10-12]. One study in Jordan shows no effect of gender at a younger age [9]. However, above 45 years of age, females seemed to have a higher OSDI score. Androgens regulate the secretory activity of the lacrimal gland [14] and their levels correlate with the signs and symptoms of DED [15]. Therefore, it was theorized that due to the lower baseline of androgens in females, the minimum required levels for the optimal functioning of the gland is reached quicker in aging women [16]. Additionally, estrogen has been shown to stimulate meibomian gland activity, which exacerbates this problem in post-menopausal women [14]. Interestingly, our study shows no statistically significant gender predilection even when corrected for age or when analyzing only those over the age of 45 years.

Age has also been shown to be a risk factor; our study agrees with this finding. There are a range of etiologic factors that have been postulated; for example, a higher incidence of comorbidities like diabetes, lowered corneal sensitivity [17], dysfunction of the lacrimal gland [18], loss of functional goblet cells [19], or the inflammatory damage of lacrimal glands [16]. Interestingly, when we graphed out the means according to the age group, we found a stark increase in the OSDI score, going from the third to fourth decades of life; thereafter, there was a steady increase.

Smoking has been a controversial risk factor for the development of DED. A few studies show an effect of smoking on the OSDI [20-21] while others disagree with this assessment [9,12]. Our study showed statistical significance while controlling for other factors. Additionally, in Figure 3, we show that for up to five years of smoking, there seems to be no effect; thereafter, a sharp increase is seen. It must be noted, however, that the baseline OSDI in smokers was higher even in those that had smoked for < one year. Therefore, it seems that there may be early effects of smoking on the ocular surface as well.

We found a significant difference in those suffering from diabetes and hypertension. Previous studies agree with this result [22-23]. It is argued that hypertension is not a direct risk factor for DED, however, antihypertensive medications contribute to the problem [22]. Encouragingly, not all drugs of hypertension have been linked to DED; some drugs like the angiotensin-converting enzyme inhibitor and receptor blockers have shown to improve the ocular surface [24]. Further research is required to understand these relationships.

There are several limitations to this study. Firstly, we did not correlate symptoms with objective signs on clinical tests. However, there are reports showing a weak association between the two; this is an area requiring further study [25]. Secondly, the Urdu version of the questionnaire was not validated. Lastly, a system of convenience sampling was employed.

## Conclusions

There is a high prevalence of DED in the population residing in Karachi, Pakistan. The exact number is hard to estimate due to various parameters that can be used. When attempting to maximize sensitivity, like other studies, we demonstrate a larger prevalence in this population when compared to others. Additionally, we demonstrate a range of factors like age, contact lens wear, ocular allergies, hypertension, and diabetes that can influence DED.
